# Mental Illness in the 2 Years Prior to Pregnancy in a Population With Traumatic Brain Injury: A Cross-Sectional Study: La maladie mentale dans les deux ans précédant une grossesse dans une population souffrant de lésion cérébrale traumatique : une étude transversale

**DOI:** 10.1177/07067437241249957

**Published:** 2024-04-25

**Authors:** Hilary K. Brown, Rachel Strauss, Kinwah Fung, Andrea Mataruga, Vincy Chan, Tatyana Mollayeva, Natalie Urbach, Angela Colantonio, Eyal Cohen, Cindy-Lee Dennis, Joel G. Ray, Natasha Saunders, Simone N. Vigod

**Affiliations:** 1 Department of Health & Society, University of Toronto Scarborough, Toronto, ON, Canada; 2 Dalla Lana School of Public Health, 7938 University of Toronto, Toronto, ON, Canada; 3 Women's College Research Institute, Women's College Hospital, Toronto, ON, Canada; 4 637532 ICES, Toronto, ON, Canada; 5 KITE Research Institute-Toronto Rehabilitation Institute, 7989 University Health Network, Toronto, ON, Canada; 6 Rehabilitation Sciences Institute, Temerty Faculty of Medicine, 7938 University of Toronto, Toronto, ON, Canada; 7 School of Medicine, Queen's University, Kingston, ON, Canada; 8 Department of Occupational Science and Occupational Therapy, Temerty Faculty of Medicine, 7938 University of Toronto, Toronto, ON, Canada; 9 Institute of Health Policy, Management and Evaluation, 7938 University of Toronto, Toronto, ON, Canada; 10 7979 Hospital for Sick Children, Toronto, ON, Canada; 11 Edwin SH Leong Centre for Healthy Children, 7938 University of Toronto, Toronto, ON, Canada; 12 Department of Pediatrics, Temerty Faculty of Medicine, 7938 University of Toronto, Toronto, ON, Canada; 13 Lawrence Bloomberg Faculty of Nursing, 7938 University of Toronto, Toronto, ON, Canada; 14 Lunenfeld-Tannenbaum Research Institute, Mount Sinai Hospital, Toronto, ON, Canada; 15 Li Ka Shing Knowledge Institute, 508783 Unity Health Toronto, Toronto, ON, Canada; 16 Department of Psychiatry, Temerty Faculty of Medicine, 7938 University of Toronto, Toronto, ON, Canada

**Keywords:** brain injuries, cohort studies, mental health, preconception care, women, lésions cérébrales, études de cohorte, santé mentale, soins préconceptionnels, femmes

## Abstract

**Objective:**

Existing studies, in mostly male samples such as veterans and athletes, show a strong association between traumatic brain injury (TBI) and mental illness. Yet, while an understanding of mental health before pregnancy is critical for informing preconception and perinatal supports, there are no data on the prevalence of active mental illness before pregnancy in females with TBI. We examined the prevalence of active mental illness ≤2 years before pregnancy (1) in a population with TBI, and (2) in subgroups defined by sociodemographic, health, and injury-related characteristics, all compared to those without TBI.

**Method:**

This population-based cross-sectional study was completed in Ontario, Canada, from 2012 to 2020. Modified Poisson regression generated adjusted prevalence ratios (aPRs) of active mental illness ≤2 years before pregnancy in 15,585 females with TBI versus 846,686 without TBI. We then used latent class analysis to identify subgroups with TBI according to sociodemographic, health, and injury-related characteristics and subsequently compared them to females without TBI on their outcome prevalence.

**Results:**

Females with TBI had a higher prevalence of active mental illness ≤2 years before pregnancy than those without TBI (44.1% vs. 25.9%; aPR 1.46, 95% confidence interval, 1.43 to 1.49). There were 3 TBI subgroups, with Class 1 (low-income, past assault, recent TBI described as intentional and due to being struck by/against) having the highest outcome prevalence.

**Conclusions:**

Females with TBI, and especially those with a recent intentional TBI, have a high prevalence of mental illness before pregnancy. They may benefit from mental health screening and support in the post-injury, preconception, and perinatal periods.

**Plain Language Title:**

Mental illness in the 2 years before pregnancy in a population with traumatic brain injury

## Plain Language Summary

Research has shown a strong association between traumatic brain injury (TBI) and mental illness. Most previous studies have been conducted in primarily male samples, like veterans and professional athletes. Understanding mental health before pregnancy is important for deciding what supports people need before and during pregnancy. However, there are no studies on the frequency of mental illness in females with TBI before a pregnancy. We examined the frequency of mental illness 2 years before pregnancy in a population with TBI, and in subgroups defined by different social, health, and injury-related characteristics, compared to those without TBI. We undertook a population-wide study of all females with and without TBI in Ontario, Canada, with a birth in 2012–2020. We used statistical models to compare these groups on the presence of mental illness in the 2 years before pregnancy, before and after accounting for social and health characteristics. We also identified subgroups with TBI according to their social (e.g., poverty), health (e.g., chronic conditions), and injury-related characteristics (e.g., cause of injury) and subsequently compared them to females without TBI on their frequency of mental illness in the 2 years before pregnancy. Forty-four percent of females with TBI had mental illness in the 2 years before pregnancy compared to 25% of those without TBI. There were 3 TBI subgroups. Females with low-income, past assault, and injuries that were described as being intentional had the highest frequency of mental illness in the 2 years before pregnancy. Females with TBI may benefit from mental health screening and support post-injury and around the time of pregnancy.

## Abstract (fr)

**Objectif:**

Il existe des études, réalisées auprès d’échantillons surtout masculins, comme des anciens combattants et des athlètes, montrant une forte association entre la lésion cérébrale traumatique (LCT) et la maladie mentale. Et pourtant, bien que la compréhension de la santé mentale avant la grossesse soit essentielle pour informer les soutiens préconceptionnels et périnataux, il n’y a pas de données sur la prévalence de la maladie mentale active avant la grossesse chez les femmes souffrant de LCT. Nous avons examiné la prévalence de la maladie mentale active ≤2 ans avant la grossesse (1) dans une population souffrant de LCT, et (2) dans des sous-groupes définis par des caractéristiques sociodémographiques, de santé, et liées à la lésion, tous se comparaient à ceux sans LCT.

**Méthode:**

Cette étude transversale dans la population a été menée en Ontario, Canada, de 2012 à 2020. La régression de Poisson modifiée a généré des ratios de prévalence ajustés (RPa) de maladie mentale active ≤2 ans avant la grossesse chez 15 585 femmes souffrant de LCT contre 846 686 sans LCT. Nous avons ensuite utilisé l'analyse des classes latentes pour identifier des sous-groupes souffrant de LCT selon nos caractéristiques sociodémographiques, de santé et liées à la lésion, et les avons subséquemment comparés aux femmes sans LCT quant à leur prévalence des résultats.

**Résultats:**

Les femmes souffrant de LCT avaient une prévalence plus élevée de maladie mentale active ≤2 ans avant la grossesse que celles sans LCT (44,1% c. 25,9%; RPa 1,46, 95% IC 1,43 à 1,49). Il y avait 3 sous-groupes LCT, la classe 1 ((faible revenu, agression passée, TBI récent décrit comme intentionnel et dû à une collision) ayant la prévalence de résultats la plus élevée.

**Conclusions:**

Les femmes souffrant de LCT, et sen particuoier celles ayant une LCT intentionnelle récente, ont une prévalence élevée de maladie mentale avant la grossesse.Elles peuvent bénéficier d’un dépistage de maladie mentale et d’un soutien dans les périodes post-traumatique, préconceptionnelle et périnatale.

## Introduction

Traumatic brain injury (TBI) is a frequent cause of disability.^1^ Approximately 2.5% of North Americans have had a TBI.^1^ One-third of persons with TBI are female,^2^ and TBI is especially common early in the reproductive years,^2^ mainly due to motor vehicle accidents and assault.^3^ TBI and mental illness frequently co-occur.^4^ Yet, while there is no significant difference between females with and without TBI in terms of their ability to conceive,^5^ little is known about the mental health of individuals with TBI going into pregnancy. This is an important issue given that preconception health impacts perinatal outcomes,^6,7^ and interventions before pregnancy mitigate risks of maternal and neonatal complications.^8,9^ Understanding the prevalence of active mental illness (i.e., current receipt of mental health care) in people with TBI before pregnancy may indicate the need for preconception interventions to optimize their mental health.

There is a large body of research, in mostly male samples such as veterans and athletes, on the co-occurrence of TBI and mental illness. The risk of TBI is increased in people with mental illness,^10,11^ and mental illness following TBI is among its most debilitating consequences, with elevated risk persisting for many years post-injury.^12–16^ Notably, there is evidence that females’ mental health after TBI may be affected more often than that of males, possibly due to their longer recovery time and greater burden of neurological symptoms, including dizziness, fatigue, and headaches.^16–18^ Yet, while an understanding of mental health before pregnancy is critical for informing preconception and perinatal supports,^9–12^ there are no data, to our knowledge, on the prevalence of active mental illness before pregnancy, overall or according to sociodemographic, health, and injury-related characteristics, among individuals with TBI.

Data on the association between TBI and active mental illness before pregnancy could show the extent of the need for trauma-informed preconception mental health screening, support, and treatment. Therefore, we examined the prevalence of active mental illness ≤2 years before pregnancy in (1) a population with TBI, and (2) subgroups with TBI defined by sociodemographic, health, and injury-related characteristics, all compared to those without TBI.

## Methods

### Study Population

We undertook a population-based cross-sectional study in Ontario, Canada (population: 14.7 million). Under Ontario's universal health-care plan, residents access medically necessary care at no direct cost. We accessed and analysed administrative data at ICES, an independent, nonprofit organization whose use of data is authorized for research under section 45 of the Ontario Personal Health Information Protection Act and does not require ethics board review. ICES datasets are linked using a unique encoded identifier and are complete and accurate.^19^ We used the Canadian Institute for Health Information (CIHI) Discharge Abstract Database (hospital admissions, including those for obstetrical deliveries); Ontario Mental Health Reporting System (admissions in facilities with mental health beds); National Ambulatory Care Reporting System (emergency department visits); Ontario Health Insurance Plan dataset (outpatient visits); Immigration, Refugees and Citizenship Canada (IRCC) Permanent Resident Database (immigration status); Registered Persons Database (dates of birth and postal code), and Census (neighbourhood characteristics).^20^ Therein, we identified all females with an obstetrical delivery conceived between 1 April 2012 and 31 March 2020 and delivered in a hospital (98% of births).^21^

### Measures

We identified TBIs recorded in ≥1 emergency department visit or hospitalization in the 10 years before pregnancy. In primary analyses, TBI was measured using an algorithm developed by the Centers for Disease Control and Prevention,^20^ which was applied to the “most responsible diagnosis” field (positive predictive value 96.9% vs. clinician-documented TBI).^22^ In additional analyses, we (1) included the exposed TBIs in any diagnostic field, which is more likely to capture polytrauma but might also capture prior TBI unrelated to the health-care encounter,^22^ and (2) used a sensitive TBI definition, which includes open wounds of the head, fracture of the orbital floor, and crushing injuries of the face and head (injuries that often accompany, but are not necessarily indicative of TBI).^23^ Females without evidence of a brain injury were the referent group.

Active mental illness prior to pregnancy was defined by ≥1 mental illness diagnosis in the 2 years before conception,^24^ captured in a visit to a general practitioner, family physician, or psychiatrist, emergency department visit, or hospitalization.^25^ We further classified active mental illness by type (i.e., mood or anxiety, psychotic, addiction, self-harm, or other mental disorder). Classification of the type of mental illness was based on all health-care encounters; as such, individuals could have more than 1 type of active mental illness. Emergency department visits for self-harm resulting in TBI were classified as part of the definition of TBI, not mental illness.

We measured several confounders based on known differences between females with and without TBI and predictors of mental illness.^5,25^ Age at conception and parity were captured in the Discharge Abstract Database-derived MOMBABY dataset. Neighbourhood income quintile was measured by linking postal codes with Census dissemination area-level (400–700 persons) income data. Rural residence was defined as living in a town or municipality with a population <10,000.^26^ Immigration status was determined from the IRCC Permanent Resident Database. History of assault, including physical or sexual assault, and other maltreatment, was captured in emergency department visits and hospital admissions in the 2 years before conception.^27^ Chronic conditions in the 2 years before conception were ascertained using the Johns Hopkins Adjusted Clinical Groups® v. 10.0^28^ and were classified as stable or unstable based on their patterns of health-care use.

In females with a TBI identified using the primary definition, we measured the (1) number of acute health-care encounters for TBI, separated by ≥24 h (1, ≥2); (2) severity of the most severe TBI, using proxies of hospitalization and discharge destination (emergency department visit only, hospitalized and discharged home, hospitalized and discharged to another facility); and (3) time since the most recent TBI (≤2, 3–10 years). Using the TBI most proximal to conception, we also measured (4) mechanism of injury (motor vehicle collision, struck by/against, falls, other),^23,29^ and (5) intent (intentional [i.e., assault/self-harm], unintentional/undetermined/other).^29^ We also planned to measure injury severity (mild, moderate, severe) using the Abbreviated Injury Scale^30^ or Glasgow Coma Scale,^31^ but the missingness of these scores was high (57.3%). We thus used this variable in sensitivity, rather than main, analyses.

### Statistical Analysis

Baseline characteristics of females with and without TBI were measured using descriptive statistics, and compared using standardized differences.^31^

We first used modified Poisson regression,^32^ with generalized estimating equations to account for clustering of births within women,^33^ to calculate unadjusted and adjusted prevalence ratios (PRs) and 95% confidence intervals (CIs) for the relation between TBI within 10 years before conception and (1) any active mental illness ≤2 years before pregnancy and (2) active mental illness by type (i.e., mood or anxiety, psychotic, addiction, self-harm, and other mental disorder). Multivariable analyses were adjusted for age, parity, income quintile, rurality, immigrant status, history of assault, and stable and unstable chronic conditions.^5,25^ Since the rate of missing data for these variables was very low (e.g., <0.4% for income quintile), a complete case analysis was conducted.

We then undertook a latent class analysis (LCA) in females with a TBI within 10 years before conception (ascertained using the primary TBI definition^20,22^). LCA is a type of finite mixture modelling that identifies homogenous subgroups within a population, defined by a set of measured variables, using the assumption that there is an unmeasured (latent) variable driving this division.^34^ Included variables were sociodemographic (age, parity, income quintile, and rurality), health (history of assault, stable and unstable chronic conditions), and injury-related characteristics (number of acute health-care encounters for TBI, injury severity, time since the most recent TBI, mechanism and intent for the most recent TBI). Since we had no a priori data to suggest an expected number of classes, we undertook an unconstrained LCA, starting with a 1-class model and increasing the number of classes until the best fit was identified.^35^ To assess model fit, we used the Akaike Information Criterion, Bayesian Information Criterion, *G*^2^ test statistic, *X*^2^ goodness of fit, and entropy.^35^ We also considered the clinical relevance of the classes.^35^ Upon identifying the best model, we described each TBI class according to its characteristics and subsequently used modified Poisson regression to estimate the PR for the association between these TBI classes and (1) any active mental illness ≤2 years before pregnancy and (2) type of active mental illness, compared to females without a TBI.

In additional analyses, we re-ran the analyses for the first and second objectives in primiparas only, given that preconception care for primiparous individuals can be slightly different than for multiparous individuals with previous perinatal experience.^6–9^ Second, we applied a specific definition of active mental illness wherein we required ≥2 outpatient physician visits, rather than ≥1. Third, we re-ran the analyses for the first objective defining the exposed group using (a) TBI recorded in any diagnostic field^22^ and (2) the more sensitive definition of TBI.^23^ Finally, we re-ran the analyses for the second objective, including the Abbreviated Injury Scale or Glasgow Coma Scale score (as available) as one of the variables in the LCA.

The analyses used SAS version 9.4 and the R poLCA package.^34^

## Results

There were 15,585 females with a TBI within 10 years before pregnancy, and 846,686 females without any history of TBI. (There were also 5,513 females with TBI recorded outside of the “most responsible diagnosis” field and 50,384 with injuries that often accompany TBI.) Females with TBI were more likely than those without TBI to be younger and primiparous and to live in low-income quintile neighbourhoods and rural regions. They were less likely to be refugees or nonrefugee immigrants and more likely to have a history of assault (Table 1).

**Table 1.** Baseline Characteristics of Females with a Traumatic Brain Injury (TBI), and Those Without any History of TBI, Within 10 Years Before Conception. Reported as No. (%) Unless Otherwise Indicated.

Baseline characteristics	TBI (*n* = 15,585)^a^	No TBI (*n* = 846,686)	Standardized difference^b^
Age (years) at conception, mean (SD)	27.2 ± 5.7	30.4 ± 5.2	0.60
15–19 years	1,340 (8.6)	21,263 (2.5)	0.27
20–24 years	4,106 (26.3)	89,249 (10.5)	0.42
25–29 years	4,827 (31.0)	240,367 (28.4)	0.06
30–34 years	3,600 (23.1)	316,357 (37.4)	0.31
35–39 years	1,448 (9.3)	150,662 (17.8)	0.25
40–44 years	249 (1.6)	27,098 (3.2)	0.10
45–49 years	15 (0.1)	1,690 (0.2)	0.03
Primiparous	8,254 (53.0)	355,374 (42.0)	0.22
Neighbourhood income quintile (Q)			
Q1 (lowest)	3,836 (24.6)	175,231 (20.7)	0.09
Q2	3,227 (20.7)	168,272 (19.9)	0.02
Q3	3,154 (20.2)	177,370 (20.9)	0.02
Q4	2,968 (19.0)	179,734 (21.2)	0.05
Q5 (highest)	2,332 (15.0)	143,994 (17.0)	0.06
Missing	68 (0.4)	2,085 (0.2)	0.03
Rural residence at conception	2,855 (18.3)	85,979 (10.2)	0.24
Immigrant status			
Long-term resident	14,230 (91.3)	623,217 (73.6)	0.48
Refugee	313 (2.0)	35,898 (4.2)	0.13
Nonrefugee immigrant	1,042 (6.7)	187,571 (22.2)	0.45
History of assault < 2 years before conception	838 (5.4)	3,258 (0.4)	0.30
Stable chronic conditions < 2 years before conception	3,464 (22.2)	190,107 (22.5)	0.01
Unstable chronic conditions < 2 years before conception	2,481 (15.9)	115,978 (13.7)	0.06

^a^ Includes individuals with a TBI recorded in “the most responsible diagnosis” field within 10 years before conception. Individuals with a TBI recorded outside of the “most responsible diagnosis” field and those with injuries that often accompany TBI (*N* = 52,134) are excluded.

^b^ Standardized differences > 0.10 indicate clinically meaningful differences between groups.

**Figure 1.**
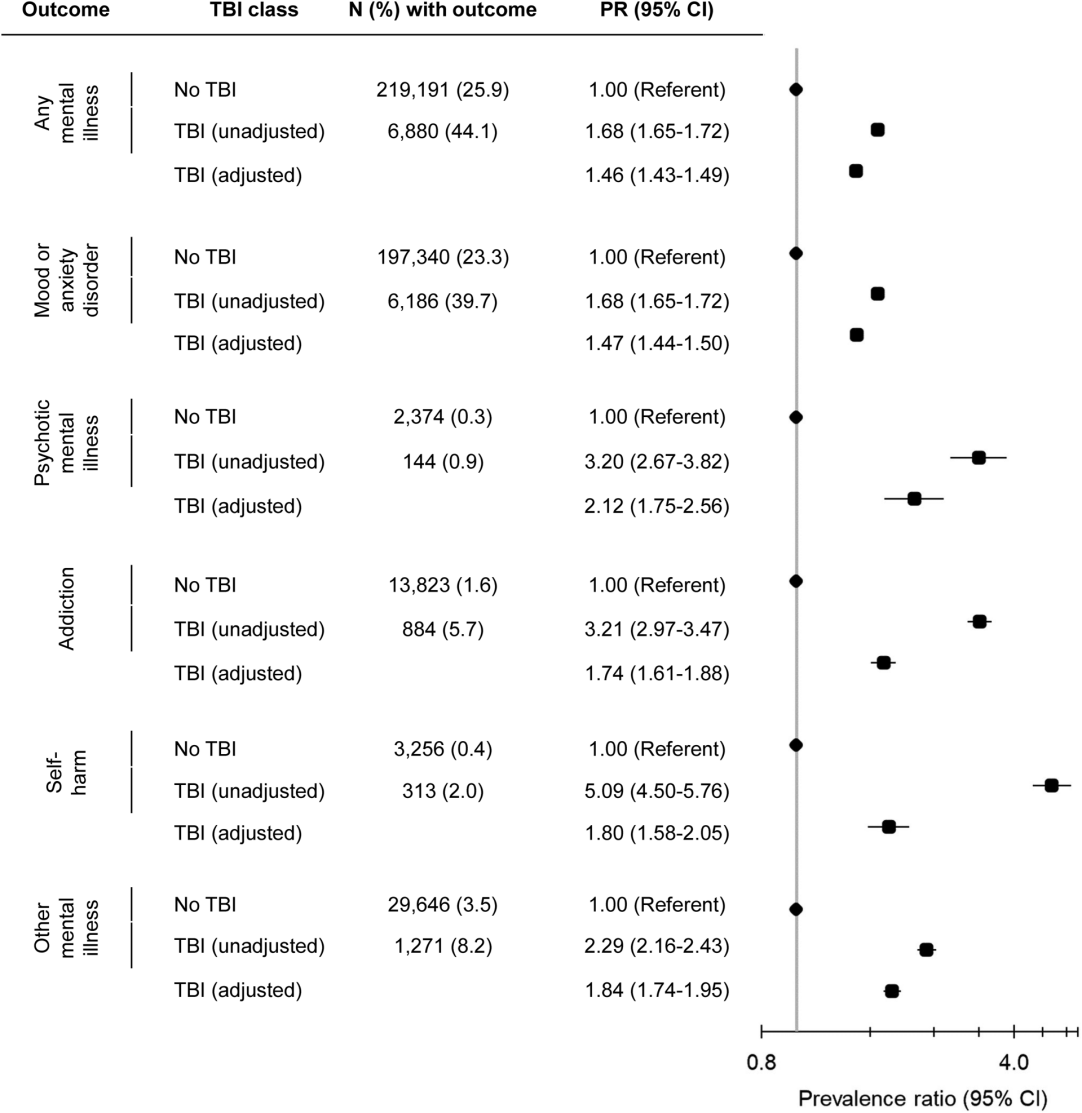
Association between traumatic brain injury (TBI) within 10 years before conception and active mental illness ≤2 years before pregnancy. TBI group includes individuals with a TBI recorded in “the most responsible diagnosis” field within 10 years before conception. Individuals with a TBI recorded outside of the “most responsible diagnosis” field and those with injuries that often accompany TBI (*N* = 52,134) are excluded. Adjusted model controls for age, parity, neighbourhood income quintile, rurality, immigrant status, history of assault, and stable and unstable chronic conditions.

Compared to females without a history of TBI, those with a TBI within 10 years before pregnancy had a higher prevalence of active mental illness ≤2 years before pregnancy (44.1% vs. 25.9%); this prevalence remained elevated after adjustment (aPR 1.46, 95% CI, 1.43 to 1.49). Similarly elevated prevalence was observed for mood or anxiety, psychotic, addiction, self-harm, and other mental disorders, with mood or anxiety disorders being the most common (Figure 1).

The LCA identified a 3-class solution (Supplemental Tables S3 and S4). In Class 1 (8.3% of females with TBI), females were mostly low-income, had a high rate of prior assault, and had a TBI in the last 2 years, with the most proximal TBI primarily described as being intentional and due to being struck by/against. In Class 2 (49.4%), females were mostly young and primiparous, with their most proximal TBI being unintentional. In Class 3 (42.3%), females were mostly in their mid-reproductive years, multiparous, higher-income, and with stable chronic conditions, with their most proximal TBI described as unintentional. Rurality, immigration status, number of acute health-care encounters for TBI, and TBI severity did not contribute to the classes (Table 2).

**Table 2.** Sociodemographic, Health, and Injury-Related Characteristics, by Class Membership, in Females with a Traumatic Brain Injury (TBI) in the 10 Years Before Conception. Reported as No. (%) Unless Otherwise Indicated.

Characteristics	Class 1 (*N* = 1,288)	Class 2 (*N* = 7,698)	Class 3 (*N* = 6,599)
Age (years)			
15–24 years	611 (47.4)	4,829 (62.7)	6 (0.1)
25–34 years	578 (44.9)	2,869 (37.3)	4,980 (75.5)
35–49 years	99 (7.7)	0 (0.0)	1,613 (24.4)
Primiparous	552 (42.9)	5,770 (75.0)	1,932 (29.3)
Neighbourhood income quintile Q1–Q2	812 (63.0)	3,923 (51.0)	2,328 (35.3)
Rural residence at conception	225 (17.5)	1,958 (25.4)	672 (10.2)
Refugee/nonrefugee immigrant	57 (4.4)	130 (1.7)	1,168 (17.7)
History of assault < 2 years before conception	484 (37.6)	322 (4.2)	32 (0.5)
Stable chronic conditions < 2 years before conception	252 (19.6)	937 (12.2)	2,275 (34.5)
Unstable chronic conditions < 2 years before conception	197 (15.3)	821 (10.7)	1,463 (22.2)
≥2 TBIs **≤ **10 years before conception	110 (8.5)	991 (12.9)	340 (5.2)
Most severe injury ≤ 10 years before conception			
A hospitalization, discharged to facility	<6	26 (0.3)	69 (1.0)
A hospitalization discharged home	50–54	288 (3.7)	309 (4.7)
Emergency department visits only	1,229 (95.4)	7,384 (95.9)	6,221 (94.3)
≤2 years since the most recent TBI	408 (31.7)	2,207 (28.7)	1,521 (23.0)
Most proximal TBI mechanism			
Motor vehicle collision	<6	1,607 (20.9)	1,967 (29.8)
Struck by/against	1,140 (88.5)	3,139 (40.8)	2,270 (34.4)
Fall	<6	2,581 (33.5)	2,101 (31.8)
Other	147 (11.4)	371 (4.8)	255–259
Missing	0 (0.0)	0 (0.0)	<6
Most proximal TBI intent			
Assault/self-harm	1,275 (99.0)	<6	<6
Unintentional/undetermined/other	13 (1.0)	7,695 (100.0)	6,592 (99.9)
Missing	0 (0.0)	<6	<6

*Note.* For binary variables, only the category of interest is reported. Values < 6 suppressed to protect patient identities.

**Figure 2.**
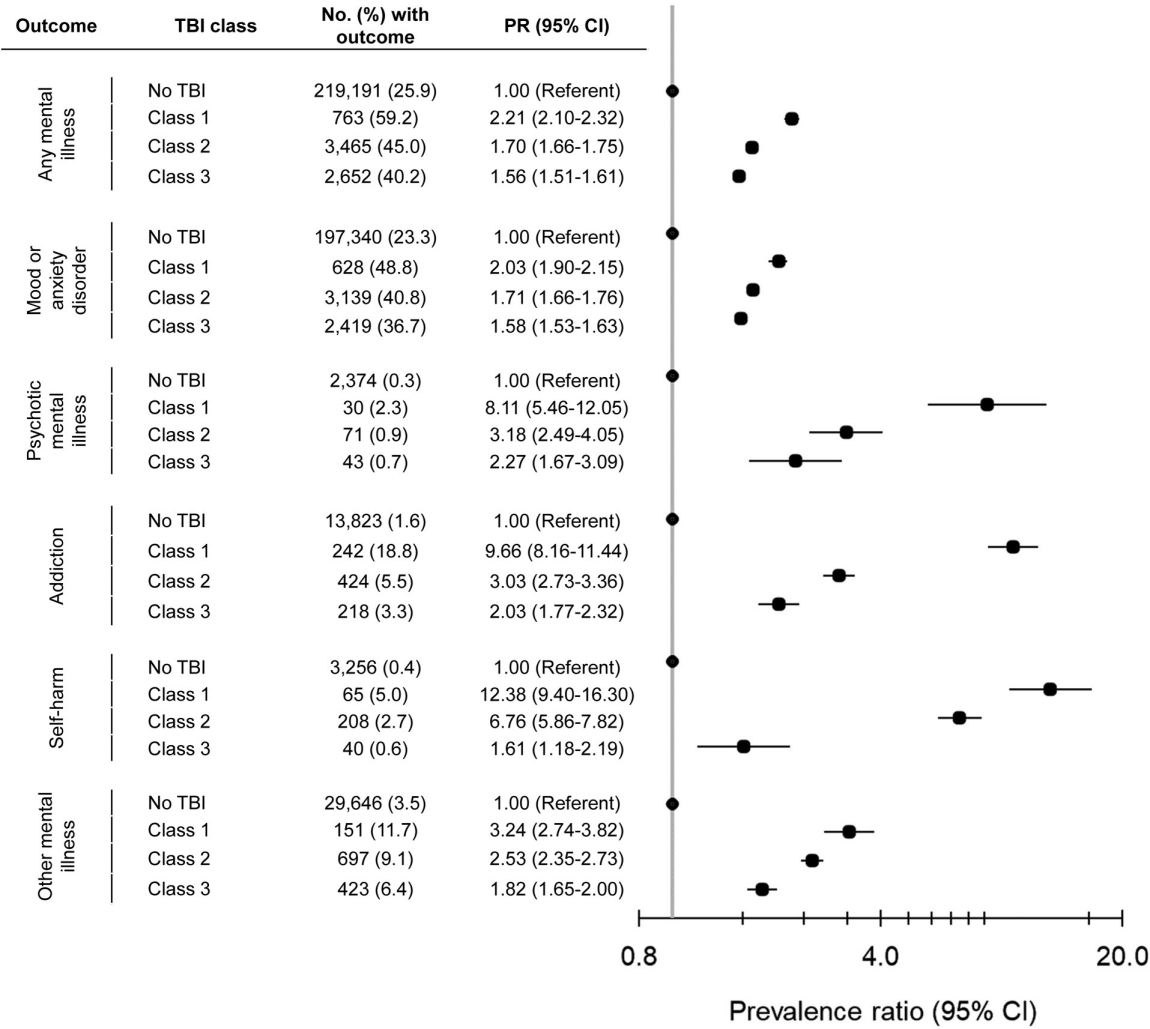
Associations between traumatic brain injury (TBI) within 10 years before conception, by TBI class, and active mental illness ≤2 years before pregnancy. Class 1: mostly low-income, high rate of past assault, TBI in the last 2 years, with the most proximal TBI primarily described as being intentional and due to being struck by/against; Class 2: mostly young and primiparous, with the most proximal TBI described as being unintentional; Class 3: mostly mid-reproductive years, multiparous, higher-income, and with chronic conditions, with most proximal TBI described as being unintentional.

When we assessed the association between these TBI classes and the prevalence of active mental illness ≤2 years before pregnancy, females in Class 1 had the greatest prevalence of any mental illness (59.2%), as well as mood or anxiety, psychotic, addiction, self-harm, and other mental disorders. Those in Class 2 had the next highest prevalence (45.0%), followed by Class 3 (40.2%). All prevalence estimates were elevated relative to females without TBI (Figure 2).

Findings were similar when the analyses were restricted to primiparas (Supplemental Tables S5 and S6), when we required ≥2 physician visits to define active mental illness ≤2 years before pregnancy (Supplemental Table S7), and when we included TBIs captured in any diagnostic field and used a more sensitive definition of TBI (Supplemental Table S8). Finally, when we included Abbreviated Injury Scale/Glasgow Coma Scale scores in the LCA, the same 3-class solution was identified (Supplemental Table S9), again with Class 1 having the highest prevalence of active mental illness (Supplemental Table S10).

## Discussion

In this large, population-based study, we found females with a TBI in the decade before pregnancy had a significantly elevated prevalence of active mental illness in the 2 years before pregnancy, with the highest prevalence in those who had assault-related and recent injuries. Findings were robust to sensitivity analyses varying the definitions of TBI and active mental illness. Given the high prevalence of mental illness in this population, this study shows the extent of need among reproductive-aged females with TBI for post-injury and preconception mental health supports.

There is a large body of literature on the relation between TBI and mental illness. This research suggests elevated risk of depression, anxiety, addiction, and suicidality in people with TBI persists up to 60 years post-injury.^16^ Yet, while fertility appears to be unaffected by TBI,^5^ only 1 study has examined the impact of TBI on mental health around the time of pregnancy.^8^ This study found women with TBI had a high rate of self-reported postpartum difficulties including depression.^8^ Mental health before pregnancy was not examined. This is an important gap in knowledge given mental health before pregnancy affects perinatal outcomes,^9,10^ with preconception mental health interventions shown to reduce risks of adverse outcomes.^11^ Our study makes an important contribution by showing an elevated prevalence of mental illness before pregnancy in females with TBI, particularly those with assault-related injuries.

As in the broader literature on TBI and mental illness, there are several possible reasons for the observed association between TBI and active mental illness before pregnancy. Mental illness may be a direct result of brain injury: for example, common areas of contusion include the temporal poles, mesial temporal structure, and orbital frontal cortices—areas of the brain known to be implicated in disorders such as depression, anxiety, and posttraumatic stress disorder.^11^ TBI is also often followed by lingering neurological symptoms, including dizziness, headaches, and fatigue, that can be debilitating and have implications for other psychosocial risk factors for mental illness, such as problems with employment and relationships.^36^ To be clear, we did not examine whether TBI preceded, or followed, the onset of mental illness. Indeed, mental illness itself is a risk factor for TBI, possibly due to problems with attention, risk-taking behaviours, and medication and medical complications that increase the risk of injury.^4^ Finally, assault is a risk factor for both TBI and mental illness in females.^3^ Indeed, in our study, the risk of active mental illness before pregnancy was highest in those with TBI who had a history of assault, and whose TBIs were intentional. It is likely that these factors contribute to a bi-directional relation between TBI and mental illness before pregnancy. Given that most research on this topic is from predominantly male populations, future research on mechanisms and risk factors in reproductive-aged females is warranted.

### Limitations

Our study included pregnancies ending in live birth or stillbirth, potentially resulting in selection bias. Future research should examine individuals who have a miscarriage or induced abortion. It is also important to note that preconception health is often defined more broadly in terms of the health of all individuals of reproductive age, regardless of their pregnancy intentions or pregnancy outcome.^6–9^ While there have been a number of studies on the association between TBI and mental illness in females broadly (regardless of age),^16–18^ future studies could also examine the association in females of reproductive age.

In primary analyses, we included TBIs recorded in the “most responsible diagnosis” field to best capture new injuries, rather than preexisting comorbidities, that is, care “for” TBI, not “with” TBI.^20,22^ This may have resulted in missing polytrauma, where another injury (e.g., spinal cord injury) was listed as the most responsible diagnosis. Nevertheless, our approach has a better positive predictive value (97%) than using all diagnostic fields (74%),^22^ and results were similar when we used all diagnostic fields. We did not capture mild TBIs that only required outpatient care or did not result in care.^37^ We also missed TBIs occurring >10 years before conception; however, our emergency department visit database began in 2002, and our 10-year lookback period ensured equal ascertainment for everyone. While we had data on injury characteristics, missingness for injury severity scores was high since this is not a required field.^38^ Also, despite our large cohort, we were unable to report on TBI due to assault and self-harm separately (though the vast majority of this category were due to assault). Finally, we were unable to measure TBI-related neurological symptoms that might be associated with mental illness risk.^16–18^

Administrative data only capture mental illness resulting in publicly funded health care, thus missing individuals with undiagnosed mental illness and those using private services. It is possible individuals with TBI experience access barriers to mental health care, resulting in under-ascertainment of mental illness. We were also unable to determine whether mental illness in the defined preconception period was new or existing^24^; however, prevalence estimates are relevant for planning screening and supports going into pregnancy.

Finally, we lacked data on covariates such as race/ethnicity as a proxy for experiences of racism. Risks for and outcomes associated with TBI are also influenced by sex and gender^5^; however, ICES data only include sex, not gender identity. Further research is necessary to explore the roles of these variables to develop appropriate mental health supports.

## Conclusions

Reproductive health, emotional health, and gender-based violence were identified by a wide range of stakeholders as research priorities at the first international workshop on women and TBI in 2010.^39^ However, health-care providers continue to identify a lack of resources on females with TBI as a barrier to care.^37^ Our data have implications for the post-injury, pregnancy planning, and perinatal periods for females with TBI. Post-injury and preconception services could include screening for mental illness, tailored mental health information, and support to optimize brain and mental health preconceptionally via better management of TBI symptoms, comorbidities, and social and environmental risk factors. Mental illness following TBI can be treatment-resistant, with more severe outcomes than among individuals without TBI.^40^ Given that nearly half of individuals with a TBI entered pregnancy with an active mental illness, our data show that health-care providers should be aware of the need for tailored treatment approaches in this population. For subgroups with experiences of assault, this may include trauma-informed perinatal mental health care that addresses intimate partner violence and related injuries.^41^ TBI in the context of intimate partner violence has been a historically overlooked area in both TBI and gender-based violence research and practice. These injuries are often not reported, and individuals may not be aware that their symptoms are due to TBI.^42^ A need for education to address this intersection has been identified,^41^ which is particularly important in the context of preconception and perinatal care, given that violence escalates around the time of pregnancy.^43^ Such efforts will require health-care providers with specialized knowledge at the intersection of TBI, trauma, and mental health, and collaborative care approaches. Since mental illness has consequences for long-term maternal and child well-being,^44^ better preconception and perinatal mental health interventions for people with TBI could have a substantial long-term, and inter-generational, impact.

## Data Access

The dataset from this study is held securely in coded form at ICES. While legal data sharing agreements between ICES and data providers (e.g., health-care organizations and government) prohibit ICES from making the dataset publicly available, access may be granted to those who meet pre-specified criteria for confidential access, available at www.ices.on.ca/DAS (email: das@ices.on.ca). The full dataset creation plan and underlying analytic code are available from the authors upon request, understanding that the computer programs may rely upon coding templates or macros that are unique to ICES and are therefore either inaccessible or may require modification.

## Disclaimers

Parts of this material are based on data and/or information compiled and provided by the Ontario Ministry of Health (MOH), the Canadian Institute for Health Information (CIHI), and Immigration, Refugees and Citizenship Canada (IRCC) current to September 2020. However, the analyses, conclusions, opinions and statements expressed in the material are those of the author(s), and not necessarily those of MOH, CIHI, or IRCC. This document used data adapted from the Statistics Canada Postal CodeOM Conversion File, which is based on data licensed from Canada Post Corporation, and/or data adapted from the Ontario Ministry of Health Postal Code Conversion File, which contains data copied under license from ©Canada Post Corporation and Statistics Canada. This does not constitute endorsement by Statistics Canada of this project. 

## Supplemental Material

sj-docx-1-cpa-10.1177_07067437241249957 - Supplemental material for Mental Illness in the 2 Years Prior to Pregnancy in a Population With Traumatic Brain Injury: A Cross-Sectional Study: La maladie mentale dans les deux ans précédant une grossesse dans une population souffrant de lésion cérébrale traumatique : une étude transversale Supplemental material, sj-docx-1-cpa-10.1177_07067437241249957 for Mental Illness in the 2 Years Prior to Pregnancy in a Population With Traumatic Brain Injury: A Cross-Sectional Study: La maladie mentale dans les deux ans précédant une grossesse dans une population souffrant de lésion cérébrale traumatique : une étude transversale by Hilary K. Brown, Rachel Strauss, Kinwah Fung, Andrea Mataruga, Vincy Chan, Tatyana Mollayeva, Natalie Urbach, Angela Colantonio, Eyal Cohen, Cindy-Lee Dennis, Joel G. Ray, Natasha Saunders and Simone N. Vigod in The Canadian Journal of Psychiatry
