# Supplementary material for: Mental Illness in the 2 Years Prior to Pregnancy in a Population With Traumatic Brain Injury: A Cross-Sectional Study: La maladie mentale dans les deux ans précédant une grossesse dans une population souffrant de lésion cérébrale traumatique : une étude transversale
Source: Can J Psychiatry. 2024 Apr 25;69(8):607–17. doi: 10.1177/07067437241249957 (PMC11298096; doi:10.1177/07067437241249957)
Supplement: sj-docx-1-cpa-10.1177_07067437241249957 - Supplemental material for Mental Illness in the 2 Years Prior to Pregnancy in a Population With Traumatic Brain Injury: A Cross-Sectional Study: La maladie mentale dans les deux ans précédant une grossesse dans une population souffrant de lésion cérébrale tr [file sj-docx-1-cpa-10.1177_07067437241249957.docx]

**Table S1. Identification of traumatic brain injury (TBI) and injury-related characteristics.**

| **Variable** | **Definition** |
| --- | --- |
| TBI (specific, most responsible diagnosis field)^23,24^ | ≥ 1 emergency department visits or hospitalizations associated with any of the following codes in the “most responsible diagnosis field” within 10 years before conception: ICD-10: S02.0, S02.1, S02.8, S02.9, S04.0, S06.0-S06.9, S07.1 |
| TBI (specific, any diagnosis field)^23,24^ | ≥ 1 emergency department visits or hospitalizations associated with any of the following codes in any diagnosis field within 10 years before conception: ICD-10: S02.0, S02.1, S02.8, S02.9, S04.0, S06.0-S06.9, S07.1 |
| TBI (sensitive, most responsible diagnosis field)^25^ | ≥ 1 emergency department visits or hospitalizations associated with any of the following codes in the most responsible diagnosis field within 10 years before conception: ICD-10: S01.0-S01.9, S02.0, S02.1, S02.3, S02.7-S02.9, S04.0, S06.0-S06.9, S07.0, S07.1, S07.8, S07.9, S09.7-S09.9, T90.1, T90.2, T90.4, T90.5, T90.8, T90.9 |
|  |  |
| Number of separate acute health care encounters for TBI | Number of health care encounters for TBI and separated by at least 24 hours between discharge time and subsequent admission time, within 10 years before conception |
| Injury severity (proxy definition for main analysis) | For the most severe TBI within 10 years before conception, measured using hospital admission and discharge destination as proxies, categorized as hospitalized and discharged to another facility (e.g., long-term care), hospitalized and discharged home, or emergency department visit only |
| Injury severity (sensitivity analysis) | For the most severe TBI within 10 years before conception, measured using the Abbreviated Injury Scale (1-2 points – mild, 3 points = moderate, ≥ 4 points = severe) and/or Glasgow Coma Scale (14-15 points = mild, 9-13 points = moderate, 3-8 points = severe); or missing |
| Time since most recent TBI acute health care encounter | Years since the most recently recorded acute health care encounter for TBI within 10 years before conception |
| Injury mechanism | For the most recent TBI health care encounter within 10 years before conception, categorized as motor vehicle collision, struck by/against, falls, other, or missing |
| Injury intent | For the most recent TBI health care encounter within 10 years before conception, categorized as assault or self-harm; unintentional, undetermined, or other (including legal interventions or war); or missing |

Abbreviation: OHIP: Ontario Health Insurance Plan; ICD-10 International Statistical Classification of Diseases and Health-Related Conditions, tenth revision.

**Table S2. Identification of active mental illness ≤ 2 years before pregnancy.**

| **Mental illness** | **Outpatient physician diagnostic and service codes** | **ICD-10 codes for hospitalizations and emergency department visits** | **DSM-IV codes for psychiatric hospitalizations** |
| --- | --- | --- | --- |
| Mood or anxiety disorders | Psychiatrist [SPEC=19] and outpatient (LOCATION: O, L, H, P) and non-lab service [substr(FEECODE,1,1) ne 'G'] OR Family physician / general practitioner / pediatrician / obstetrician [SPEC=00, SPEC=20, SPEC=26] and mental illness or addiction diagnosis code [DXCODE] and outpatient (LOCATION: O, L, H, P) and non-lab service [substr(FEECODE,1,1) ne 'G'] OR pediatrician [SPEC=26] and undefined location (LOCATION=U) and MHA diagnostic code [DXCODE] and fee code (FEECODE=K122 or K123 or K704), where DXCODES = 296, 300, 309, 311 | Before 2016/17: DX10CODE1 F30-F34, F38-F43, F48.8, F48.9, F53.0, F93.1-F93.2; 2016/17-present: DX10CODE1 F06.3, F06.4, F30-F34, F38-F43, F45.2, F53.0, F63.3, F93.0-F93.2, F94.0-F94.2 | Before 2016/17: AXIS1_DSM4CODE_DISCH1 = 296.x (all 296 codes), 300, 300.0x, 300.2x, 300.3x, 300.4x, 301.13, 308.3x, 309.0x, 309.24, 309.28, 309.3x, 309.4x, 309.8x, 309.9x, 311; Provisional: 6, 7, 15. 2016/17-2018/19: DSM5CODE_DISCH1 = 293.83, 293.84, 296.x (all 296 codes), 300, 300.0x, 300.2x, 300.3x, 300.4x, 300.7x, 301.13, 308.3x, 309, 309.0x, 309.21, 309.24, 309.28, 309.3x, 309.4x, 309.81, 309.89, 309.9x, 311.x, 312.39, 313.23, 313.89, 625.4, 698.4x, Provisional = 3-7; 2019/20 to present: ICD10CMCODE_DISCH1=F06.3, F06.4, F06.8, F31-F34, F40.0-F40.2, F41.0, F41.1, F41.8, F41.9, F42.2-F42.4, F42.8, F42.9, F43.0-F43.2, F43.8, F43.9, F45.2, F63.3, F91.4, F94.2, F93.0, F94.0-F94.2; Provisional = 3-7 |
| Psychotic disorders | Psychiatrist [SPEC=19] and outpatient (LOCATION: O, L, H, P) and non-lab service [substr(FEECODE,1,1) ne 'G'] OR Family physician / general practitioner / pediatrician / obstetrician [SPEC=00, SPEC=20, SPEC=26]and mental illness or addiction diagnosis code [DXCODE] and outpatient (LOCATION: O, L, H, P) and non-lab service [substr(FEECODE,1,1) ne 'G'] OR pediatrician [SPEC=26] and undefined location (LOCATION=U) and MHA diagnostic code [DXCODE] and fee code (FEECODE=K122 or K123 or K704), where DXCODES = 295, 297, 298 | DX10CODE1 F20 (excluding F20.4), F22-F25, F28-F29, F53.1 | Before 2016/17: AXIS1_DSM4CODE_DISCH1 = 295.x (all 295 codes), 297.x (all 297 codes), 298.x (all 298 codes); Provisional = 5; 2016/17-2018/19: DSM5CODE_DISCH1 = 293.81, 293.82, 295.x (all 295 codes), 297.x (all 297 codes), 298.x (all 298 codes), Provisional = 2; 2019/20 to present: ICD10CMCODE_DISCH1=F20.81, F20.9, F22, F23, F25, F06.0-F06.2, F28, F29; Provisional = 2 |
| Addiction | Psychiatrist [SPEC=19] and outpatient (LOCATION: O, L, H, P) and non-lab service [substr(FEECODE,1,1) ne 'G'] OR Family physician / general practitioner / pediatrician / obstetrician [SPEC=00, SPEC=20, SPEC=26]and mental illness or addiction diagnosis code [DXCODE] and outpatient (LOCATION: O, L, H, P) and non-lab service [substr(FEECODE,1,1) ne 'G'] OR pediatrician [SPEC=26] and undefined location (LOCATION=U) and MHA diagnostic code [DXCODE] and fee code (FEECODE=K122 or K123 or K704), where DXCODES = 291, 292, 303, 304 | DX10CODE1 F10-F19, F55 | Before 2016/17: AXIS1_DSM4CODE_DISCH1 = 291.x (all 291 codes, excluding 291.82), 292.x (all 292 codes, excluding 292.85), 303.x (all 303 codes), 304.x (all 304 codes), 305.x (all 305 codes), PROVDX_DSM4CODE_ADM1: 4; 2016/17-2018/19: DSM5CODE_DISCH1 = 291.x (all 291 codes), 292.x (all 292 codes), 303.x (all 303 codes), 304.x (all 304 codes), 305.x, Provisional = 16; 2019/20 to present: ICD10CMCODE_DISCH1=F10-F19, Z72.0; Provisional = 16 |
| Self-harm | -- | DX10CODE2-10 X60-X84, Y10-Y19, Y28 (self-harm resulting in TBI is coded as an exposure, not an outcome) | -- |
| Other | Psychiatrist [SPEC=19] and outpatient (LOCATION: O, L, H, P) and non-lab service [substr(FEECODE,1,1) ne 'G'] OR Family physician / general practitioner / pediatrician / obstetrician [SPEC=00, SPEC=20, SPEC=26]and mental illness or addiction diagnosis code [DXCODE] and outpatient (LOCATION: O, L, H, P) and non-lab service [substr(FEECODE,1,1) ne 'G'] OR pediatrician [SPEC=26] and undefined location (LOCATION=U) and MHA diagnostic code [DXCODE] and fee code (FEECODE=K122 or K123 or K704), where DXCODES = 301, 302, 306, 307, 309, 313-315 | DX10CODE1 All other F06-F99 from above not included in other categories, and excluding IDD diagnoses | All other OMHRS from above not included in other categories, and excluding IDD diagnoses |

Abbreviation: ICD-10 International Statistical Classification of Diseases and Health-Related Conditions, tenth revision; DSM-IV Diagnostic and Statistical Manual of Mental Disorders, 4^th^ revision

**Table S3.** **Indicators of fit and predicted probability of class membership for latent class models with 1 to 7 classes.**

| **Measures of Model Fit** | | | | | | | |
| --- | --- | --- | --- | --- | --- | --- | --- |
|  | **1 class** | **2 classes** | **3 classes** | **4 classes** | **5 classes** | **6 classes** | **7 classes** |
| **AIC:** | 215732.4 | 211898.8 | 209834.4 | 208660.9 | 207866.2 | 207432.3 | 207103.0 |
| **BIC:** | 215900.8 | 212243.2 | 210354.9 | 209357.4 | 208738.8 | 208480.9 | 208327.7 |
| **G^2^:** | 15760.6 | 11881.0 | 9770.6 | 8551.1 | 7710.5 | 7230.5 | 6855.2 |
| **X^2^:** | 99957165.0 | 63329025.0 | 6117297.0 | 44070066.0 | 4308746.0 | 659635.9 | 687974.0 |
| **Entropy:** | N/A | 0.998552 | 0.999095 | 0.999287 | 0.999388 | 0.999452 | 0.999496 |
|  | | | | | | | |
| **Predicted Probability of Class Membership** | | | | | | | |
|  | **1 class** | **2 classes** | **3 classes** | **4 classes** | **5 classes** | **6 classes** | **7 classes** |
| **1 class** | 1.0000 | -- | -- | -- | -- | -- | -- |
| **2 classes** | 0.0877 | 0.9123 | -- | -- | -- | -- | -- |
| **3 classes** | 0.0826 | 0.4939 | 0.4234 | -- | -- | -- | -- |
| **4 classes** | 0.0272 | 0.4137 | 0.5031 | 0.0560 | -- | -- | -- |
| **5 classes** | 0.0271 | 0.4024 | 0.0574 | 0.0367 | 0.4764 | -- | -- |
| **6 classes** | 0.0267 | 0.4507 | 0.3331 | 0.0940 | 0.0368 | 0.0580 | -- |
| **7 classes** | 0.4167 | 0.3247 | 0.0564 | 0.0268 | 0.0844 | 0.0320 | 0.0590 |

Abbreviations: AIC = Akaike Information Criterion, BIC = Bayesian Information Criterion; G^2^ = G-squared test statistic; X^2^ = Chi-squared goodness of fit.

**Table S4. Predicted probabilities of class membership for sociodemographic, health, and injury-related characteristics in the latent class analysis model with a 3-class solution.^a^**

| **Variable** | **Predicted probability of class membership** | | |
| --- | --- | --- | --- |
|  | **Class 1** | **Class 2** | **Class 3** |
|  | **0.09** | **0.50** | **0.41** |
| Age (years) |  |  |  |
| 15-24 years | **0.48** | **0.60** | **0.02** |
| 25-34 years | **0.44** | **0.40** | **0.73** |
| 35-49 years | 0.07 | 0.00 | 0.25 |
| Primiparous | **0.43** | **0.67** | **0.39** |
| Neighbourhood income quintile Q1-Q2 | **0.63** | **0.50** | **0.36** |
| Rural residence at conception | 0.17 | 0.25 | 0.11 |
| Refugee/non-refugee immigrant | 0.04 | 0.02 | 0.17 |
| History of assault < 2 years before conception | **0.37** | 0.04 | 0.01 |
| Stable chronic conditions < 2 years before conception | 0.20 | 0.15 | **0.31** |
| Unstable chronic conditions < 2 years before conception | 0.15 | 0.12 | 0.21 |
| ≥ 2 TBIs **≤** 10 years before conception | 0.09 | 0.12 | 0.06 |
| Most severe injury ≤ 10 years before conception |  |  |  |
| A hospitalization, discharged to facility | 0.00 | 0.00 | 0.01 |
| A hospitalization, discharged home | 0.04 | 0.04 | 0.04 |
| Emergency department visit only | **0.96** | **0.95** | **0.95** |
| ≤ 2 years since most recent TBI | 0.32 | 0.28 | 0.24 |
| Most proximal TBI mechanism |  |  |  |
| Motor vehicle collision | 0.00 | 0.23 | 0.28 |
| Struck by/against | **0.88** | **0.40** | **0.35** |
| Fall | 0.00 | **0.33** | **0.33** |
| Other | 0.12 | 0.05 | 0.04 |
| Missing | 0.00 | 0.00 | 0.00 |
| Most proximal TBI intent |  |  |  |
| Assault / self-harm | **0.92** | 0.00 | 0.00 |
| Unintentional / undetermined / other | 0.08 | **1.00** | **1.00** |
| Missing | 0.00 | 0.00 | 0.00 |

^a^ Values > 0.30 are considered meaningful (in **bold**).

**Table S5. Sociodemographic, health, and injury-related characteristics, by class membership, in primiparous females with a traumatic brain injury (TBI) within 10 years before conception.**

| **Injury variables** | **Predicted probabilities** | | | **Prevalence** | | |
| --- | --- | --- | --- | --- | --- | --- |
|  | **Class 1**  **(0.07)** | **Class 2**  **(0.46)** | **Class 3**  **(0.47)** | **Class 1**  **(N=553)** | **Class 2**  **(N=3,789)** | **Class 3**  **(N=3,912)** |
| Age (years) |  |  |  |  |  |  |
| 15-24 years | **0.64** | **0.71** | 0.04 | 345-350 | 3,227 (85.2) | < 6 |
| 25-34 years | **0.33** | 0.29 | **0.80** | 184 (33.3) | 562 (14.8) | 3,368 (86.1) |
| 35-49 years | 0.04 | 0.00 | 0.16 | 22 (4.0) | 0 (0.0) | 540-545 |
| Neighbourhood income quintile Q1-Q2 | **0.60** | **0.50** | **0.33** | 331 (59.9) | 1,937 (51.1) | 1,363 (34.8) |
| Rural residence at conception | 0.17 | 0.25 | 0.09 | 94 (17.0) | 1,156 (30.5) | 215 (5.5) |
| Refugee/non-refugee immigrant | 0.04 | 0.02 | 0.15 | 23 (4.2) | 97 (2.6) | 499 (12.8) |
| History of assault < 2 years before conception | **0.42** | 0.04 | 0.00 | 234 (42.3) | 190-195 | < 6 |
| Stable chronic conditions < 2 years before conception | 0.17 | 0.14 | 0.24 | 96 (17.4) | 525 (13.9) | 905 (23.1) |
| Unstable chronic conditions < 2 years before conception | 0.13 | 0.12 | 0.18 | 73 (13.2) | 441 (11.6) | 665 (17.0) |
| ≥ 2 TBIs **≤** 10 years before conception | 0.11 | 0.14 | 0.06 | 62 (11.2) | 627 (16.5) | 180 (4.6) |
| Most severe injury ≤ 10 years before conception |  |  |  |  |  |  |
| A hospitalization, discharged to facility | 0.00 | 0.01 | 0.00 | < 6 | 10-15 | 30 (0.8) |
| A hospitalization, discharged home | 0.03 | 0.04 | 0.04 | 15-20 | 150 (4.0) | 155 (4.0) |
| Emergency department visit only | **0.97** | **0.95** | **0.96** | 533 (96.4) | 3,627 (95.7) | 3,727 (95.3) |
| ≤ 2 years since most recent TBI | **0.38** | 0.26 | **0.32** | 209 (37.8) | 1,249 (33.0) | 989 (25.3) |
| Most proximal TBI mechanism |  |  |  |  |  |  |
| Motor vehicle collision | 0.00 | 0.28 | 0.22 | 0 (0.0) | 790 (20.8) | 1,078 (27.6) |
| Struck by/against | **0.92** | **0.37** | **0.41** | 510 (92.2) | 1,585 (41.8) | 1,461 (37.3) |
| Fall | 0.00 | **0.32** | **0.32** | 0 (0.0) | 1,226 (32.4) | 1,236 (31.6) |
| Other | 0.08 | 0.04 | 0.05 | 43 (7.8) | 188 (5.0) | 135-140 |
| Missing | 0.00 | 0.00 | 0.05 | < 6 | 0 (0.0) | < 6 |
| Most proximal TBI intent |  |  |  |  |  |  |
| Assault / self-harm | **0.91** | 0.00 | 0.00 | 548 (99.1) | < 6 | < 6 |
| Unintentional / undetermined / other | 0.10 | **1.00** | **1.00** | < 6 | 3,785-3,790 | 3,909 (99.9) |
| Missing | 0.00 | 0.00 | 0.00 | < 6 | 0 (0.0) | < 6 |

AIC: 97050.9; BIC: 97486.1; G^2^: 4274.3; X^2^: 24006665.0; Entropy: 0.998508285.

**Table S6. Associations between traumatic brain injury (TBI) within 10 years before conception, overall and by TBI class, and any active mental illness ≤ 2 years before pregnancy, among primiparas only.**

|  | **No. (%) with outcome** | **Unadjusted prevalence ratio (95% CI)** | **Adjusted prevalence ratio**  **(95% CI)^b^** |
| --- | --- | --- | --- |
| **Any mental illness** |  |  |  |
| No TBI (N=355,374) | 90,233 (25.4) | 1.00 (Referent) | 1.00 (Referent) |
| TBI (N=8,254)^a^ | 3,662 (44.4) | 1.75 (1.70-1.79) | 1.48 (1.44-1.52) |
|  |  |  |  |
| **Any mental illness** |  |  |  |
| No TBI (N=355,374) | 90,233 (25.4) | 1.00 (Referent) | 1.00 (Referent) |
| TBI Class 1 (N=553) | 332 (60.0) | 2.36 (2.21-2.53) | -- |
| TBI Class 2 (N=3,789) | 1,821 (48.1) | 1.89 (1.83-1.96) | -- |
| TBI Class 3 (N=3,192) | 1,509 (38.6) | 1.52 (1.46-1.58) | -- |

^a^ Includes individuals with a traumatic brain injury recorded in “the most responsible diagnosis” field within 10 years before conception. Individuals with traumatic brain injury recorded outside of the “most responsible diagnosis” field and those with injuries that often accompany TBI (N=24,498) are excluded.

^b^ Adjusted model controls for age, parity, neighbourhood income quintile, rurality, immigrant status, history of assault, and stable and unstable chronic conditions.

**Table S7. Associations between traumatic brain injury (TBI) within 10 years before conception and any active mental illness ≤ 2 years before pregnancy, using the specific definition of mental illness.**

|  | **No. (%) with outcome** | **Unadjusted prevalence ratio (95% CI)** | **Adjusted prevalence ratio**  **(95% CI)^b^** |
| --- | --- | --- | --- |
| **Any mental illness** |  |  |  |
| No TBI (N=846,686) | 158,524 (18.7) | 1.00 (Referent) | 1.00 (Referent) |
| TBI (N=15,585)^a^ | 5,736 (36.8) | 1.91 (1.87-1.95) | 1.58 (1.54-1.61) |
|  |  |  |  |
| **Any mental illness** |  |  |  |
| No TBI (N=846,686) | 158,524 (18.7) | 1.00 (Referent) | 1.00 (Referent) |
| TBI Class 1 (N=1,288) | 683 (53.0) | 2.66 (2.50-2.82) | -- |
| TBI Class 2 (N=7,698) | 2,950 (38.3) | 1.96 (1.90-2.02) | -- |
| TBI Class 3 (N=6,599) | 2,103 (31.9) | 1.70 (1.64-1.76) | -- |

^a^ Includes individuals with a traumatic brain injury recorded in “the most responsible diagnosis” field within 10 years before conception. Individuals with traumatic brain injury recorded outside of the “most responsible diagnosis” field and those with injuries that often accompany TBI (N=52,134) are excluded.

^b^ Adjusted model controls for age, parity, neighbourhood income quintile, rurality, immigrant status, history of assault, and stable and unstable chronic conditions.

**Table S8. Associations between traumatic brain injury (TBI) within the 10 years before conception and any active mental illness ≤ 2 years before pregnancy, using alternative definitions of TBI.**

|  | **No. (%) with mental illness** | **Unadjusted prevalence ratio (95% CI)** | **Adjusted prevalence ratio**  **(95% CI)^b^** |
| --- | --- | --- | --- |
| **Any mental illness** |  |  |  |
| No TBI (N=846,686) | 219,191 (25.9) | 1.00 (Referent) | 1.00 (Referent) |
| TBI (specific, any diagnostic field) (N=17,335)^a^ | 7,680 (44.3) | 1.69 (1.66-1.72) | 1.46 (1.44-1.49) |
|  |  |  |  |
| **Any mental illness** |  |  |  |
| No TBI (N=846,686) | 219,191 (25.9) | 1.00 (Referent) | 1.00 (Referent) |
| TBI (sensitive, most responsible diagnosis field) (N=62,206)^b^ | 25,436 (40.9) | 1.55 (1.53-1.56) | 1.36 (1.35-1.38) |

^a^ Includes individuals with a traumatic brain injury recorded in any diagnostic field using the specific definition of TBI. Individuals with injuries that often accompany traumatic brain injury (N=50,384) are excluded.

^b^ Includes individuals with a traumatic brain injury recorded in “the most responsible diagnosis” field within the 10 years before conception using the sensitive definition of TBI. Individuals with traumatic brain injury recorded outside of the “most responsible diagnosis” field (N=5,513) are excluded.

^c^ Adjusted model controls for age, parity, neighbourhood income quintile, rurality, immigrant status, history of assault, and stable and unstable chronic conditions.

**Table S9. Sociodemographic, health, and injury-related characteristics (including Abbreviated Injury Scale/Glasgow Coma Scale score), by class membership, in females with a traumatic brain injury (TBI) within 10 years before conception.**

| **Injury variables** | **Predicted probabilities** | | | **Prevalence** | | |
| --- | --- | --- | --- | --- | --- | --- |
|  | **Class 1**  **(0.09)** | **Class 2**  **(0.49)** | **Class 3**  **(0.42)** | **Class 1**  **(N=1,292)** | **Class 2**  **(N=7,189)** | **Class 3**  **(N=7,014)** |
| Age (years) |  |  |  |  |  |  |
| 15-24 years | 0.48 | 0.61 | 0.02 | 613 (47.4) | 4,826 (67.1) | 7 (0.1) |
| 25-34 years | 0.44 | 0.39 | 0.73 | 580 (44.9) | 2,363 (32.9) | 5,484 (77.2) |
| 35-49 years | 0.07 | 0.00 | 0.24 | 99 (7.7) | 0 (0.0) | 1,613 (22.7) |
| Primiparous | 0.43 | 0.67 | 0.39 | 552 (42.7) | 5,240 (72.9) | 2,462 (34.7) |
| Neighbourhood income quintile Q1-Q2 | 0.63 | 0.50 | 0.36 | 816 (63.2) | 3,873 (53.9) | 2,374 (33.4) |
| Rural residence at conception | 0.17 | 0.25 | 0.11 | 225 (17.4) | 1,957 (27.2) | 673 (9.5) |
| Refugee/non-refugee immigrant | 0.04 | 0.02 | 0.17 | 57 (4.4) | 129 (1.8) | 1,169 (16.5) |
| History of assault < 2 years before conception | 0.37 | 0.04 | 0.01 | 488 (37.8) | 316 (4.4) | 34 (0.5) |
| Stable chronic conditions < 2 years before conception | 0.20 | 0.15 | 0.31 | 254 (19.7) | 929 (12.9) | 2,281 (32.1) |
| Unstable chronic conditions < 2 years before conception | 0.15 | 0.12 | 0.20 | 197 (15.2) | 781 (10.9) | 1,503 (21.2) |
| ≥ 2 TBIs **≤** 10 years before conception | 0.09 | 0.12 | 0.06 | 110 (8.5) | 1,007 (14.0) | 324 (4.6) |
| Most severe injury ≤ 10 years before conception |  |  |  |  |  |  |
| A hospitalization, discharged to facility | 0.00 | 0.00 | 0.01 | < 6 | 23 (0.3) | 72 (1.0) |
| A hospitalization, discharged home | 0.04 | 0.04 | 0.04 | 50-55 | 279 (3.9) | 318 (4.5) |
| Emergency department visit only | 0.96 | 0.95 | 0.95 | 1,233 (95.4) | 6,887 (95.8) | 6,714 (94.5) |
| Most severe injury ≤ 10 years before conception based on AIS/GCS |  |  |  |  |  |  |
| Severe (3-8 points) | 0.01 | 0.01 | 0.01 | 10-15 | 46 (0.6) | 88 (1.2) |
| Moderate (9-13 points) | 0.00 | 0.01 | 0.01 | < 6 | 67 (0.9) | 82 (1.2) |
| Mild (14-15 points) | 0.38 | 0.44 | 0.38 | 493 (38.2) | 3,326 (46.3) | 2,543 (35.8) |
| Unknown | 0.61 | 0.55 | 0.59 | 782 (60.5) | 3,750 (52.2) | 4,391 (61.8) |
| ≤ 2 years since most recent TBI | 0.32 | 0.28 | 0.24 | 410 (31.7) | 2,170 (30.2) | 1,556 (21.9) |
| Most proximal TBI mechanism |  |  |  |  |  |  |
| Motor vehicle collision | 0.00 | 0.23 | 0.28 | < 6 | 1,607 (20.9) | 1,967 (29.8) |
| Struck by/against | 0.88 | 0.40 | 0.35 | 1,140 (88.5) | 3,139 (40.8) | 2,270 (34.4) |
| Fall | 0.00 | 0.33 | 0.33 | < 6 | 2,581 (33.5) | 2,101 (31.8) |
| Other | 0.12 | 0.05 | 0.04 | 147 (11.4) | 371 (4.8) | 255-260 |
| Missing | 0.00 | 0.00 | 0.00 | 0 (0.0) | 0 (0.0) | < 6 |
| Most proximal TBI intent |  |  |  |  |  |  |
| Assault / self-harm | 0.91 | 0.00 | 0.00 | 1,275 (99.0) | < 6 | < 6 |
| Unintentional / undetermined / other | 0.09 | 1.00 | 1.00 | 13 (1.0) | 7,695 (100.0) | 6,592 (99.9) |
| Missing | 0.00 | 0.00 | 0.00 | 0 (0.0) | < 6 | < 6 |

AIC: 233952.3; BIC: 234541.6; G^2^: 15210.6; X^2^: 45178106.0; Entropy: 0.998990586.

**Table S10. Association between traumatic brain injury (TBI) within 10 years before conception, by TBI class (including Abbreviated Injury Scale/Glasgow Coma Scale score), and any active mental illness ≤ 2 years before pregnancy.**

|  | **No. (%) with outcome** | **Unadjusted prevalence ratio (95% CI)** |
| --- | --- | --- |
| **Any mental illness** |  |  |
| No TBI (N=846,686) | 219,191 (25.9) | 1.00 (Referent) |
| TBI Class 1 (N=1,292) | 766 (59.3) | 2.21 (2.10-2.32) |
| TBI Class 2 (N=7,189) | 3,304 (46.0) | 1.74 (1.69-1.79) |
| TBI Class 3 (N=7,104) | 2,810 (39.6) | 1.53 (1.48-1.58) |

^a^ Includes individuals with a traumatic brain injury recorded in “the most responsible diagnosis” field within 10 years before conception. Individuals with traumatic brain injury recorded outside of the “most responsible diagnosis” field and those with injuries that often accompany TBI (N=52,134) are excluded.
